# Kyphotic deformity of the lumbar spine due to a monostotic fibrous dysplasia of the second lumbar vertebra: a case report and its surgical management

**DOI:** 10.1007/s00701-020-04531-2

**Published:** 2020-08-17

**Authors:** Anna Stocsits, Sara Lener, Pierre Pascal Girod, Anto Abramovič, Claudius Thomé, Sebastian Hartmann

**Affiliations:** Department of Neurosurgery, Medical University Innsbruck, Anichstrasse 35, A-6020 Innsbruck, Austria

**Keywords:** Monostotic fibrous dysplasia, Deformity, Lumbar spine, Minimally invasive

## Abstract

Monostotic fibrous dysplasia (MFD) of the lumbar spine represents an exceedingly rare lesion. A 26-year-old patient presented with a progressive osteolytic lesion of the vertebral body L2 and the diagnosis of MFD. A minimally invasive left-sided eXtreme Lateral Interbody Fusion (XLIF) approach with resection of the vertebral body L2 with placement of a mesh cage was performed. No complications were observed perioperatively and the symptoms rapidly improved. Minimally invasive piecemeal resection with a combined dorsolateral approach showed a favorable clinical and radiological outcome and seems to be a safe and reliable technique for MFD.

## Background

Monostotic fibrous dysplasia (MFD) of the spine represents a rare disease entity of the lumbar spine [3, 5, 6, 19]. In 1938, fibrous dysplasia (FD) was first characterized as a rare developmental disorder by Lichtenstein et al. [11]. In 1942, Lichtenstein and Jaffe described single (monostotic) or multiple (polyostotic) bone involvement [10]. Nowadays, FD is known as an uncommon mosaic disease, in which normal bone gets replaced by fibro-osseous tissue. This bony remodification leads to a weakened osseous matrix prone to fractures and deformity with corresponding pain and functional impairment [8]. FD represents 5 to 7% of benign bone tumors [4]. The etiology of the disease has been linked to activating mutations of the GNAS gene that encodes the α subunit of stimulatory G protein (Gsα) located at 20q13.2-13.3 [19]. The monostotic form of the disease is much more common (80%) than the polyostotic form (20%) [4]. Extra-osseous involvement of polyostotic fibrous dysplasia (PFD) including the skin (abnormal skin pigmentation) with accompanied precocious puberty, hyperthyroidism, and other extra-skeletal abnormalities presenting as the so called McCune–Albright syndrome was described [8]. MFD can affect every part of the skeleton, but is commonly found in jaw bones, ribs, femur, and tibia [14]. Spinal manifestation is rare, but more likely to be observed in the polyostotic form (7 to 24%) than in MFD (1 to 5%) [3, 17]. Especially lumbar spine involvement is extremely rare [1, 3, 6, 9, 12, 15, 19]. We represent the second case of MFD of the second lumbar vertebra and its surgical management.

## Case report

### History and presentation

**Fig. 1:**
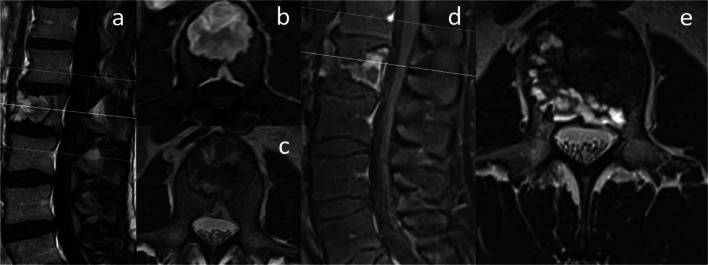
**a**, **b** Preoperative sagittal and axial T1-weighted and contrast-enhanced MRI dated 2016 revealing the osteolytic mass with medullary expansion, ground-glass matrix, narrow zone of transition, and a marginal sclerosis of L2, mainly affecting the anterior column. **c** Preoperative axial T2-weighted MRI dated 2016. **d**, **e** Preoperative sagittal and axial T1-weighted and contrast-enhanced MRI dated 2018 showing a progression of the osteolytic mass with a compression fracture of the upper endplate with increasing central cover plate recess at the posterior margin, affecting the anterior and middle spinal column leading to instability. No compression of nervous structures was observed

**Fig. 2:**
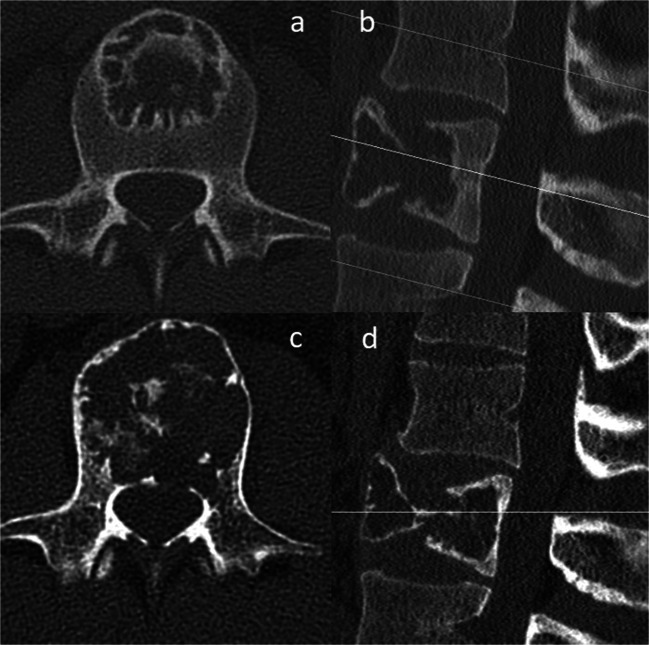
**a**, **b** Preoperative sagittal and axial computed tomography (CT) scans (2016) of L2. **c**, **d** Progression of the cystic cavitation with involvement of the anterior and posterior edge (2018)

In November 2018, a 26-year-old young man with deep-seated pain in the upper lumbar spine and the diagnosis of juvenile fibrous dysplasia of the vertebral body L2 was sent to our outpatient clinic. In 2016, a biopsy of the suspected vertebral body L2 was performed at an outside institution with the diagnosis of FD. Images of the whole body did not revealed any other lesions, so that the diagnosis of a MFD was confirmed. In the clinical course, multiple magnetic resonance images (MRI) were performed and showed a progressive osteolytic mass with medullary expansion, ground-glass matrix, narrow zone of transition, and marginal sclerosis of the second lumbar vertebral body (Fig. 1a–e). Additionally, an impression fracture of the upper endplate as well as a progression of a cystic cavitation at the posterior margin and, consequently, affecting the anterior and middle spinal column leading to instability was observed. No compression of the spinal canal or the exiting nerve roots was present. Computed tomography (CT) scans showed the described cystic cavitation with involvement of the anterior and posterior edge of the vertebral body L2 (Fig. 2a–d). Additionally, the plain lateral radiograph (x-ray) of the entire spine demonstrated incipient kyphosis at this level. Conservative treatment with pain killers and physiotherapy for more than 24 months had been performed without improvement. Due to the radiological deterioration and progressive symptoms, an indication for surgery was seen.

### Operative procedure

Surgical treatment, namely piecemeal resection of the lesion, was performed via a minimally invasive eXtreme Lateral Interbody Fusion (XLIF) approach on the left side to perform a partial resection of L2. The twelfth rib was partially resected. For vertebral body replacement, a SynMesh-Cage (DePuy-Synthes Spine, PA, USA), filled with bone harvested from the iliac crest and the resected twelfth rib, was performed. This was supplemented with demineralized bone matrix (GRAFTON™ DBM, Medtronic, Dublin, Ireland). In the same anesthesia, the patient was rotated into the prone position and a minimally invasive posterior transpedicular stabilization from L1 to L3 including lordosing screws at L2 was performed (Viper Prime; DePuy-Synthes Spine, PA, USA).

### Postoperative course

**Fig. 3:**
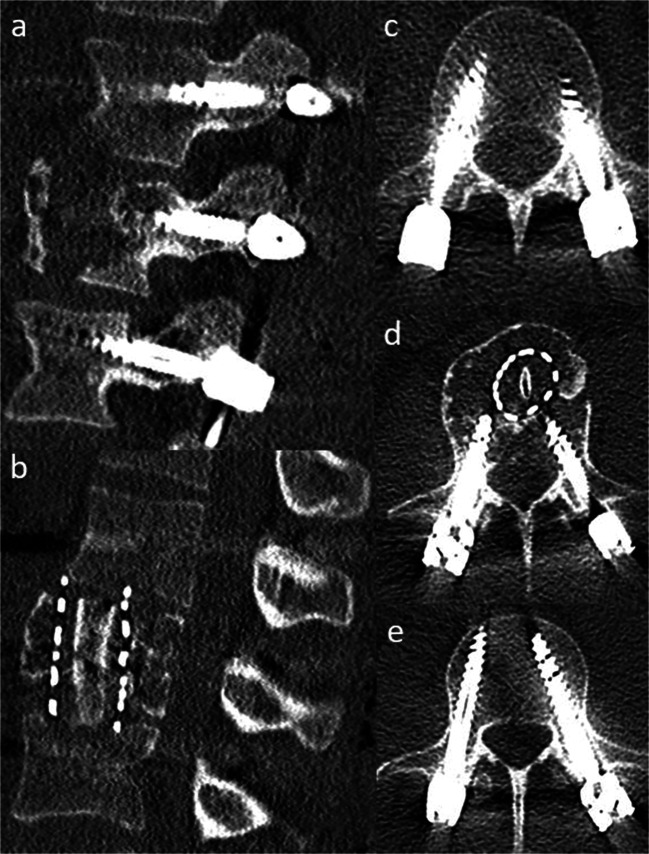
**a**, **b** Sagittal CT scans after a 360° approach with partial corpectomy, mesh cage, rib graft, and pedicle screw instrumentation. **c**–**e** Postoperative axial CT scans demonstrating pedicle screws in L1 (**c**), L2 (**d**), and L3 (**e**)

The patient did not suffer from any perioperative complications. Postoperative CT scan depicted correct positioning of the ventrodorsal instrumentation (Fig. 3a–f). The histopathological evaluation confirmed the radiological and bioptical diagnosis of fibrous dysplasia with no evidence of malignancy. At 12-month follow-up, the patient was satisfied with the surgical result and suffered from only slight stress-related pain (NRS 1-2). The postoperative x-ray did not show loss of correction.

## Discussion

FD is a rare mosaic disorder in which normal bone is replaced by fibro-osseous tissue. FD can occur throughout the skeleton; however, most often the long bones, ribs, and craniofacial bones are affected [4]. Although the monostotic form of the disease is diagnosed significantly more frequently (80% vs. 20%), to the best of our knowledge, only 17 cases of monostotic involvement of the lumbar spine have been described in the literature [12, 15, 19]. In the reported cases, the severity of the disease, the local involvement of the lumbar vertebra, and especially the treatment of FD vary widely [6, 12, 15, 19]. Concerning the vertebral level, Yu et al. published a case report with a review of the literature in 2014, which revealed that the third lumbar vertebra (L3) was most commonly affected. Mostly the vertebral body (12 cases) and rarely the spinal process (1 case) were affected [19]. Proschek et al. argued that the frequent involvement of the vertebral body is due to the large amount of cancellous bone [14]. This, in turn, supports the hypothesis that FD starts in the vertebral body and then spreads out posteriorly via the pedicles [14, 19]. In the presented case report, the vertebral body L2 of a 26-year-old patient was affected, which seems to be extremely rare [12]. According to the literature, the peak age for FD affects the 3rd life decade without sex predilection [4, 5, 19]. Due to increasing instability in the affected motion segment of the spine, the symptoms of MFD can range from pain, deformity, and fracture to nerve entrapment. The main symptom is localized pain, but in some cases, the bony remodification of the vertebral body and its consecutive changes of the affected motion segment may lead to radiculopathy [19]. Radiologically, the characteristics of spinal FD are similar to extraspinal lesions, such as medullary expansion, ground-glass matrix, narrow zone of transition, and a variable degree of marginal sclerosis [3, 12, 19]. CT and MRI are helpful to exclude an aggressive lesion with cortical destruction and soft tissue extension. Importantly, for differential diagnosis, hemangioma, giant cell tumor, and aneurysmal bone cyst, which occur more frequently, may mimic FD [12, 19]. Appropriate treatment of MFD depends on the extent of the bony lesion and its associated instability. Usually FD lesions are benign and as a consequence do not necessarily require surgical therapy. Nevertheless, sarcomatous transformation of FD has been reported [7, 15, 16]. Due to the rarity of the disease and its common benignity, the treatment varies from conservative management including biopsy and observation with analgetic therapy to surgical interventions [14, 15, 17]. Additionally, a conservative treatment approach using bisphosphonate pamidronate has been described, as it inhibits osteoclast activity and leads to an improvement in pain with no effect on the natural history of the disease [2, 4, 15, 19]. Even in case of instability, this treatment choice might be an option in older, high-risk patients. In the present case with progressive spinal deformity with instability and involvement of the anterior and middle column associated with failed conservative treatment, the surgical option to stabilize and realign the lumbar spine was chosen. Operative treatment is indicated to confirm the diagnosis by histology without prior bioptic intervention accompanied with progressive deformities and to prevent pathologic fractures in the event of incipient instability [4, 19]. Regarding surgical treatment in MFD, the opinions differ from aggressive resection and stabilization to just curettage with bone-grafting [3, 19]. The published cases used a traditional open approach [6, 9, 11, 18], which involves long incisions and tissue dissection, which can lead to more muscle trauma, blood loss, and associated pain [13]. With this in mind, a minimally invasive XLIF approach to perform a partial resection of the vertebral body combined with a posterior minimally invasive transpedicular stabilization from L1 to L3 was performed with favorable clinical and radiological outcome. More extensive or even en bloc resections are not needed. Due to fact that the patient of the presented case did not suffer from central canal compression or from nerve root irritation with accompanied radiating pain, a posterior decompression was not necessary.

## Conclusion

The authors present a rare case of MFD of the second lumbar vertebra and its unique treatment with a combined minimally invasive dorsolateral approach to prevent progressive deformity. The favorable clinical and radiological outcome in the follow-up period emphasizes the safe and well-practicable surgical treatment to resolve the symptoms in a MFD patient with progressive spinal deformity.
